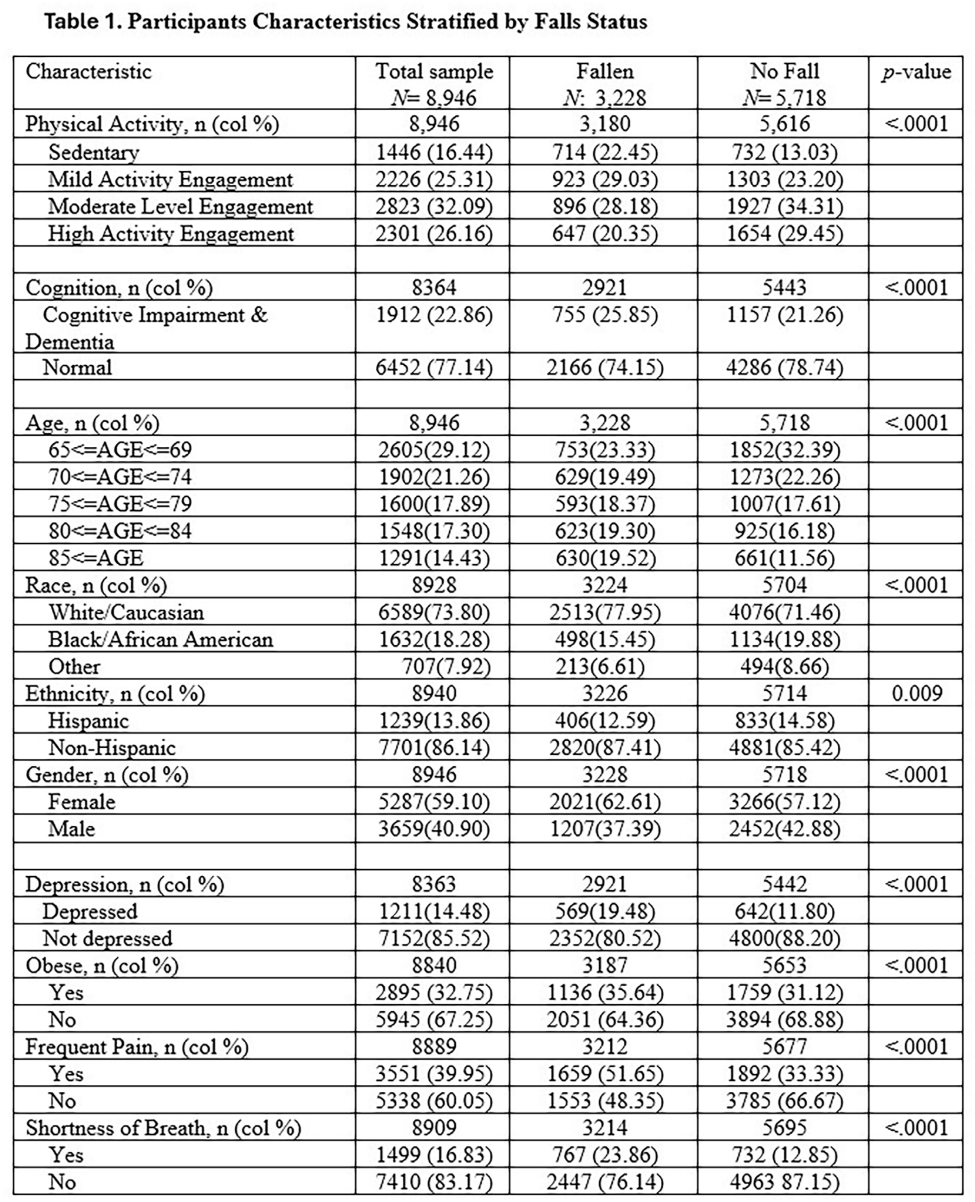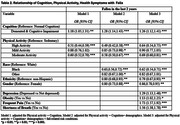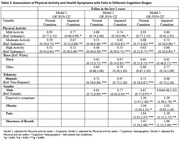# Physical Activity, Comorbid Health Symptoms and Falls in Diverse Older Americans at Different Cognitive Stages

**DOI:** 10.1002/alz.095655

**Published:** 2025-01-09

**Authors:** Ayse Malatyali, Lisa Ann Kirk Wiese, Tom Cidav, Ladda Thiamwong, Rui Xie

**Affiliations:** ^1^ University of Central Florida, 12201 Research Parkway, Suite 300 Orlando, FL USA; ^2^ C.E. Lynn College of Nursing, Florida Atlantic University, Boca Raton, FL USA; ^3^ U.S. Department of Veterans Affairs, Philadelphia, PA USA; ^4^ University of Central Florida, Orlando, FL, 32826, FL USA; ^5^ University of Central Florida, Orlando, FL USA

## Abstract

**Background:**

Engagement in regular physical activity is considered one of the most effective interventions in fall prevention, while physical inactivity is often associated with an increased risk of falls. However, research on the relationship between physical activity and falls in older adults at different cognitive stages is limited. This study describes the association of physical activity and comorbid health symptoms (depression, obesity, pain, and shortness of breath) with falls in persons with normal cognition (NC) and impaired cognition (IC).

**Method:**

We conducted a cross‐sectional study on Health and Retirement Study participants aged 65 and above (N = 8,943) using datasets from the 2020 interviews. Our measures include the 27‐point cognition scale, physical activity questionnaire, Center for Epidemiological Studies Depression scale, and health questionnaire.

**Result:**

Main logistic regression results (Table 2) showed that participants with IC were 26% more likely to fall than those with NC. Participants with moderate and high physical activity engagement were less likely to fall (31% and 36%, respectively) than physically inactive participants. However, there was no significant association between mild activity and physical inactivity. In our stratified models (Table 3), each physical activity level was significantly associated with a decreased risk of falls in the IC group, with high activity engagement showing the strongest effect. We observed similar significant trends in the NC group for high and moderate activity engagement but not mild activity. Black participants were 37% to 39% less likely to fall in the NC and IC groups. Although the risk of falling was 29% less in Hispanic participants in the NC group, the association of ethnicity and falls was non‐significant in the IC group. Women had a higher risk of falls than men; the effect of this relationship was marginal in the IC group and significant in the NC group. Depression, pain, and shortness of breath were significantly associated with falls in both cognition groups, whereas the relationship between obesity and falls was significant only in the NC group.

**Conclusion:**

These findings inform fall prevention programs for older persons at different cognitive stages, considering ethnic, racial, and gender disparities and comorbid symptoms.